# Quality of Adherence to the ARRIVE Guidelines in the Material and Methods Section in Studies Where Swine Were Used as Surgical Biomodels: A Systematic Review (2013–2018)

**DOI:** 10.3390/ani9110947

**Published:** 2019-11-11

**Authors:** Jilma Alemán-Laporte, Gilbert Alvarado, Mariana SA Garcia-Gomes, Ana Tada Fonseca Brasil Antiorio, Marco Zúñiga-Montero, Claudia Madalena Cabrera Mori

**Affiliations:** 1 Department of Pathology, School of Veterinary Medicine and Animal Science, University of São Paulo, 05508-270 São Paulo, Brazil; mari-aranha@hotmail.com (M.S.G.-G.); anatbrasil@usp.br (A.T.F.B.A.); claumori@gmail.com (C.M.C.M.); 2 Laboratorio de Docencia en Cirugía y Cáncer (DCLab), University of Costa Rica, 10501 San José, Costa Rica; marzm10@gmail.com; 3 Laboratory of Experimental and Comparative Pathology (LAPECOM), Biology School, University of Costa Rica, 10501 San José, Costa Rica; gilbert.alvarado@ucr.ac.cr

**Keywords:** pigs, ARRIVE guidelines, surgery, analgesia, anesthesia

## Abstract

**Simple Summary:**

Through a systematic review of reports where swine were used as animal biomodels for testing or researching new surgical techniques, we sought to determine the quality of the report of the methodologies carried out on the basis of the ARRIVE guidelines (Animal Research: Reporting of In Vivo Experiments) in a total of 108 studies from 2013 to 2018. In a large percentage of the articles, the information presented in the methodology of the studies showed incomplete data according to the ARRIVE guidelines recommendations for reporting the use of animals. There was a strong focus on descriptions of surgical techniques; however, sample size calculation, description of maintenance conditions, animal handling, and anesthetic and pain management protocols used were not very detailed. This could lead to the inability of others to replicate the described experiments. For this reason, we encourage authors to implement the ARRIVE guidelines to improve the quality of scientific reports and ensure animal welfare.

**Abstract:**

Over the last two decades, pigs have become animal biomodels widely used for the investigation and practice of surgical techniques because of their great physiological and anatomical similarities to humans. Even though many of these studies must be carried out later in humans, the description of basic information is limited, making exact repetitions of the reported experimental methods impossible. In this review, 108 studies from 2013 to 2018 were considered to determine the quality of adherence to the ARRIVE guidelines in the reports of the methodologies. The majority of the studies lacked the details recommended in the ARRIVE guidelines regarding data directly related to the welfare of animals undergoing surgery and those about anesthetic protocols and analgesics. Information related to sample size calculation and housing and husbandry conditions was also very limited. We believe that the ARRIVE guidelines are an excellent tool for good-quality reporting. We encourage scientists to consistently use them as a tool to improve the quality of their scientific reports and, consequently, ensure animal welfare.

## 1. Introduction

Swine have become popular animal models for preclinical trials for medical research because of their size and anatomical and physiological similarity to humans. For this reason, these animals are widely used for research on physiopathology and new surgical techniques. Over the last 20 years, swine have replaced dogs as the general surgical model for both training and research [1].

A scientific and moral argument is that if pigs are used to ‘model’ human beings undergoing surgery, then they should receive the equivalent standards of perioperative care humans would; however, most bioscience journals provide little or no guidance on what information to report when describing animal research, and many details are omitted [2]. Unfortunately, this might be a contributing reason why researchers have been increasingly unable to replicate the positive results from animal studies in the clinical trials that have followed [3]. Ideally, scientific publications should present enough information to allow a knowledgeable reader to understand what, why, and how experiments were done and to assess the reliability and validity of findings [4]. Omitting essential information might lead to scientific and ethical concerns and does not facilitate the reproducibility of the experimental conditions [5].

The National Centre for the Replacement, Refinement and Reduction of Animals in Research (NC3Rs), a UK government-sponsored scientific organization, has led an initiative to produce guidelines for reporting animal research. In 2010, the Animal Research: Reporting of In Vivo Experiments (ARRIVE) guidelines were published [5] to address the growing concerns with poor experimental design and lack of transparent reporting of in vivo experiments in published literature. These guidelines consist of a checklist of 20 categories that provide all the information that researchers should include in scientific publications using animals [6].

Recent publications focused on the quality of reporting revealed very little improvement in reporting standards since the guidelines were introduced [7,8]. Research with pigs has also presented minimum information published on topics about perioperative care (anesthetic protocols and pain alleviation) [2]. Bradbury et al. (2016) found that reporting postoperative pain management in studies was remarkably low, reflecting either under-reporting or under-use of analgesics. For this reason, a systematic review was performed to evaluate the quality of reports on research in surgeries where swine were used as biomodels. We decided to analyze only the materials and methods section of each article, with a special emphasis on anesthetic and analgesic procedures, because this section describes the procedures that have a direct impact on animal welfare.

## 2. Materials and Methods

### 2.1. Search Strategy

A literature review was performed of articles that were published from January 2013 to December 2018. An internet search was performed using PubMed, Google Scholar, Scopus, and Science Direct as electronic databases. The keywords used were: Swine OR Pig OR Minipig AND Surgery.

### 2.2. Inclusion and Exclusion Criteria

Articles included had these characteristics:

- Original articles published in English
- Use of swine models in vivo
- Studies that included surgery as an experimental procedure
- Studies published between 2013 and 2018. This period was selected to sample the recent biomedical literature (for the last 5 years), considering a suitable period for the ARRIVE guidelines to be implemented
- Studies that described painful experimental surgical procedures like skin incision, craniotomies, thoracotomies, laparotomies, laparoscopies, dental surgeries, and orthopedic surgeries

Review articles, commentaries, or communications were excluded. Studies without in vivo experiments were also excluded.

### 2.3. Evaluation of Publication Quality

We used the ARRIVE guidelines to analyze the articles, focusing on the “Material and Methods” section to evaluate the degree of compliance of publications with these guidelines.

We established a score based on 3 levels for the evaluation of the categories of the guidelines. They were defined as follows: Score 0: not mentioned, total absence of any type of information, Score 1: unclear/not complete, items not mentioned completely in the category assess, Score 2: adequate/clear, complete information for all items corresponding to the category evaluated.

### 2.4. Statistics

Data were extracted from the articles and presented into tables, and the information of each ARRIVE’s category, subcategory, and evaluated item was calculated and expressed as percentage.

Forty-three subcategories or items were evaluated as the levels of greater detail indicated for the ARRIVE guidelines. The frequency for each year was calculated and expressed in percentage according to the maximum and minimum percentage presented for each subcategory or item.

## 3. Results

### 3.1. Study Selection

**Figure 1:**
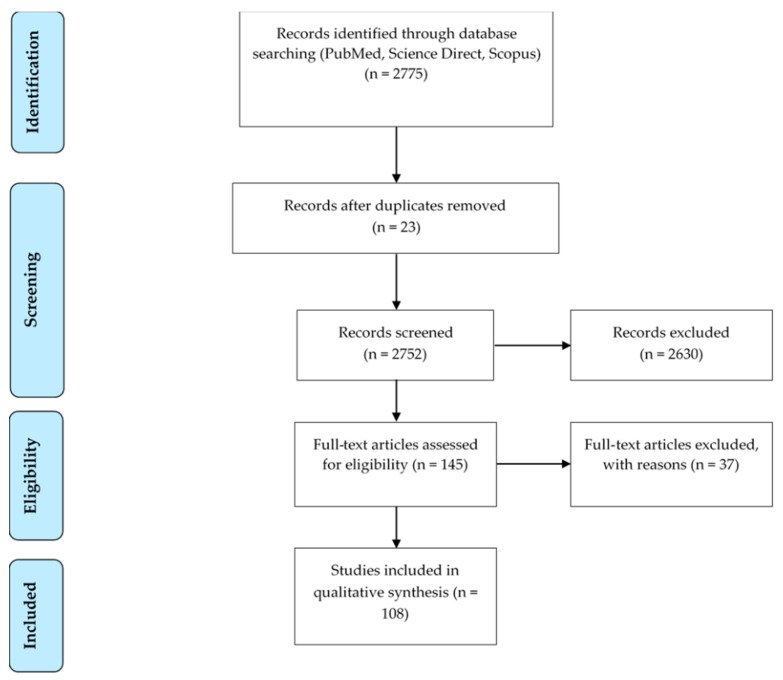
Flow diagram summarizing the search strategy.

From a total of 2775 articles, 145 articles were eligible by the analyses of the title and abstract. After this, a complete review of each article was done, and 37 articles were excluded for the following reasons: surgical procedures not included in the aforementioned list (n = 18), surgical procedures just cited without description (n = 6), procedures performed on dead animals (n = 6), non-original articles (n = 4), surgical procedures were not performed (n = 3) (Figure 1). Finally, a total of 108 publications (Supplementary Information S1) in 81 different journals fulfilled the inclusion criteria required for this systematic review. PRISMA guidelines [9] were used to design this search strategy (Figure 1).

### 3.2. Information Analyzed in Each Article

In general, the studies included some information related to the methodology used with the animals. However, most of the literature lacked quality on the basis of the ARRIVE guidelines, even though 27 of the journals consulted encouraged other publishers to use the ARRIVE guidelines. All categories of reporting information in all articles were unclear or incomplete, except the category of experimental outcomes, for which most of the journals gave very little or no information (Table 1).

Of all articles analyzed, those published in 2014 and 2015 provided more information in the “Material and Methods” section (42% and 35%, respectively, of the 43 subcategories/items presented maximum mean of report for the years considered). Articles published in 2013, 2016, and 2018 presented less information (around 30% of the 43 subcategories/items presented minimum mean of report for the years considered) (Table 2).

The most reported subcategories/items were the following: surgical procedure (100%), total number of animals used (94%), and approval by an ethical committee (92%). Several subcategories/items were not mentioned in all articles (e.g., time of the day, 0%) or little mentioned (e.g., choice of a specific anesthetic, route, and dose, 1%; bedding material, 3%; environmental enrichment, 3%; an explanation of how the number of animals was determined, 3%) (Table 2).

### 3.3. Surgery and Anesthesia

Most reported surgical procedures in the articles were laparotomies, orthopedic surgeries, and thoracotomies (Table 3).

### 3.4. Anesthesia

The drug used for anesthesia was reported in 81% of articles; however, the doses of each of these drugs were only recorded in 71% of articles, and the route of administration in 68%. Most commonly used drugs were ketamine (in 58.3% of articles), isoflurane (in 44.4% of articles), xylazine (in 31.5% of articles), and midazolam (in 25% of articles). These drugs were used alone or in combination with other drugs, reaching up to 55 different anesthetic protocols.

### 3.5. Analgesia

Intraoperative or postoperative analgesia was only reported in 41% of articles. Doses of the analgesic drugs used were reported in 32% of articles, and the route chosen for administration was reported in only 24% of articles. Fentanyl (reported in 14.8% of articles) and lidocaine (reported in 13.9% of articles) were the drugs used for intraoperative analgesia in most studies. The most used drugs in postoperative analgesia were buprenorphine (reported in 11.1% of articles) and meloxicam (reported in 8.3% of articles).

## 4. Discussion

The “Materials and Methods” section of research papers should provide basic information about how research was performed. Comprehensive reporting is essential to understand how investigations were undertaken, to interpret findings properly [7], and to allow the reproduction of results if the same methodology will be applied in other studies. The information analyzed in this systematic review shows that many of these studies would not be reproducible, considering that important details were omitted.

After examining articles from 2013 (three years after the creation and implementation of the ARRIVE guide), we found that the report of all data describing the used methodology according to the ARRIVE guidelines did not improve through the years, even though 27 of the 81 journals consulted request the authors to use these guidelines. The outcomes obtained resemble those found by Barker [10] et al. in 2014. These authors made an analysis of papers published in *PLOS* and *Nature* journals and observed very little improvement in reporting standards since the ARRIVE guidelines were published in 2010. In fact, they observed in this review a better report of the methods in the years 2014 and 2015 (although they were incomplete in most cases), but then the quality of the reports decreased (2016 was the year with less reported data, followed by 2018 and 2013). The reproducibility of these studies in similar studies can thus be affected [2,3,4,5,6,7,8,9,10,11].

The high percentage of reports of ethical review permissions is an indication that projects were previously reviewed by ethical institutional committees to ensure the welfare of the animals; however, many articles did not indicate the protocol numbers or the guides on which their protocols were based. These data should allow tracking the approved protocols and the guides that justify the care provided to the animals used in each investigation [12].

The report of study design showed a relative increase in the number of subcategories of experimental and control groups and experimental units; however, a detailed description of the methods to avoid bias was undermentioned (it was present only in 27% of articles). The lack of randomization and blinding can affect the scientific validity because biased—both conscious and unconscious—factors with no relation to the biological action can influence the results; therefore, mentioning the way an experiment was done is critical [13].

Considering the experimental procedures, the journals did not require important data such as the time at which the experiments were performed, in all the articles. Probably, because of the scope of these studies focused on surgical procedures’ description, this factor may not have been considered. The circadian cycle might change animal physiology, leading to different results, which makes it important to provide the time of day the experiments were done to ensure experiment reproducibility [14]. Other little detailed data were the choice of specific medications, doses, and administration routes in swine undergoing surgery. According to its pharmacokinetics and pharmacodynamics, each medication presents a different effect on animal physiology, which may alter the results, so justifying their use is essential. Similarly, different routes of administration may lead to different absorption levels, and the effect generated may produce differences in the results obtained, so their choice should be well justified as well [15].

The least mentioned data in the category of experimental animals were the source, sex, and race of the animals. These data should be mandatory in any report of experimental procedures using animals because genetic and/or hormonal characteristics of the animals might influence the results. Most articles also inadequately detailed information about housing and husbandry. Although the studies were not specifically on animal welfare issues, these data must be mentioned, so that other researchers can reproduce the same conditions. The experimental animals should not be unnecessarily stressed and should be kept under appropriately controlled conditions. Poor animal welfare is likely to result in poor science [7].

Most of the studies reported the number of animals; however, almost none reported the statistical method used to calculate this number. Determining the sample size by power size or simple calculations helps to design animal research with an appropriate number of animals to detect important biological effects. Omission in reporting means potentially flaws on a research [16].

The statistical methods were not mentioned in 24% of the studies. This section should not be omitted because it shows the way data were analyzed. Likewise, much of this information reported in journals was not detailed, and thus, its lack raises doubts on the validity of the statistical methods chosen to evaluate the results. While focusing on technically challenging research and on generating innovative science, many journals fail to ensure compliance with the basic standards of experimental design and data analysis. One solution to this problem is to have an additional statistical review of submitted manuscripts (as it is often done by journals in health sciences). In addition, learned societies might suggest methods of analysis of standard outcomes and data reporting to their members [10].

All the surgical procedures generate pain at different intensities, according to tissue invasiveness [17]. In this review, the reported surgeries mostly generated moderate to severe pain. Thoracotomy and orthopedic surgeries were the most invasive. On the contrary, dentistry was considered slightly less invasive, and skin incisions and craniotomies the least invasive procedures [18]. That is why the use of effective anesthetic and analgesic protocols is essential to ensure the welfare of the animals, decreasing pain and stress.

In this review, the drugs used to anesthetize the animals were generally reported at a high level of (81%). The great variety of anesthetic protocols and administered doses showed the absence of standardization for these protocols. This may be due to the type of procedure developed and the local availability of products. However, it was reported that ketamine, which is classified as a N-methyl D-Aspartate (NMDA) antagonist that causes a dissociative anesthesia, [19], was the most used drug in combination with other medications for both induction and anesthetic maintenance. Isoflurane was the second anesthetic administered alone or in combination with other drugs, probably because it allows the maintenance of the animal’s unconsciousness in a simple and long-lasting manner.

On the other hand, the under reporting of analgesia is a concern because it reveals an inadequate management of pain in pigs, which has not being improved over the years (2014 was the year with the highest number of mentions of the drugs used, which appeared only in 56% of the articles). These results are like those of the Bradbury study, in which the authors also conducted a review of pain management in swine in articles from 2012 to 2014. The need to control pain, particularly in animals used in research, is not only for ethical reasons, but also because pain side effects may occur in the used animals [18]. Along with surgical stress, pain leads to an endocrine response, which could generate massive physiological changes that could alter the quality of the results [20]. In addition, all cases were biomedical studies that sought to extrapolate results to humans. Conditions should be the same as those used in a human patient, otherwise, the results cannot be compared. Coulter and colleagues [21] found that papers reporting ethical approvals were also more likely to report about systemic analgesic administration than those that did not. Furthermore, standards of ethical review differ widely between countries. The lack of reporting data may be due to the fact that the objectives of these articles were not related to animal pain. Failure to provide adequate postoperative analgesia undermines the three Rs principles, not complying with refinement—which seeks to improve experimental procedures to avoid suffering and pain of the animal—, replacement—individual animals should be replaced by alternative methods—, or reduction—less animals should be employed, using of a more powerful study design [2].

Another factor is that the selected drugs should be the best choice for a good pain management in the experiments. The few articles examined in this review that reported analgesics looked for adequate options for pain management. Fentanyl (the most reported intraoperative analgesic in the articles examined) is a strong opioid that can generate excellent analgesia. Lidocaine, on the other hand, is a local anesthetic widely used to generate local anesthesia in dental and orthopedic surgeries. For postoperative analgesia, buprenorphine (an opioid agonist drug that has been shown to have a longer duration of action in pigs compared to other opioid drugs) [22] was the most commonly used drug, and meloxicam (a nonsteroidal anti-inflammatory drug (NSAID) that preferentially inhibits cyclooxygenase-2 and has demonstrated potent analgesic and anti-inflammatory activities) was the second most used drug. However, few articles mentioned how postoperative pain was evaluated, which does not allow the reader to verify if the protocol used was the most appropriate for the type of surgery performed. The development of a pain-scoring system in pigs, together with the mandatory description of pain management in submitted articles, would contribute to improve laboratory pig welfare [2].

## 5. Conclusions

Our review reveals poor reporting in studies of surgical procedures in swine, which has worsened over the years, especially for data directly related to the welfare of animals undergoing surgery, such those regarding anesthetic protocols and analgesics. We believe that the ARRIVE guidelines are an excellent tool to achieve high-quality reports. However, their underutilization may be due to a lack of commitment on the part of many authors to use this guide and to the fact that many journals do not require that this guide be followed, especially by those whose main objective is not animal welfare. Increasing the methodology details can lead to increased article length, and many journals have a maximum word limit. However, details can be added as Supplementary Materials [17]. We encourage authors and journals to make use of this guide to improve the quality of scientific reporting and, consequently, ensure animal welfare.

## Figures and Tables

**Table 1:** Scores used to assess the quality of the reported methods in selected articles describing surgical procedures in pigs (based on the ARRIVE guidelines).

Category	0	1	2
1. Ethical Statement	6%	80%	14%
2. Study Design	21%	53%	26%
3. Experimental procedures	0%	100%	0%
4. Experimental animals	3%	90%	7%
5. Housing and Husbandry	43%	57%	0%
6. Sample size	7%	90%	3%
7. Allocating animals to experimental groups	19%	74%	7%
8. Experimental outcomes	95%	N/A	5%
9. Statistical methods	37%	49%	14%

0: No mentioned 1: Unclear/Not complete 2: Adequate/Clear. N/A: not applicable.

**Table 2:** Information obtained from the revision of categories, subcategories, and evaluated items, based on the ARRIVE guidelines.

Category	Subcategory	Items	Mean (%)	Max. (%, Year)	Min. (%, Year)
1. Ethical Statement	1.1. Ethical review permissions	1.1.1. Refers to guidelines	52	69 (2014)	35 (2017)
		1.1.2. Approved by ethical committee	92	100 (2016)	81 (2014)
		1.1.3. Protocol number	20	57(2018)	17 (2013)
2. Study Design	2.1. Number of experimental and control groups		71	88 (2014)	57 (2018)
	2.2. Steps taken to minimize the effects of subjective bias		27	40 (2016)	11 (2013)
	2.3. The experimental unit		79	94 (2014)	57 (2018)
3. Experimental procedures	3.1. How	3.1.1. Anesthesia drugs	81	94 (2015)	71 (2017)
		3.1.2. Dose of anesthesia	71	83 (2015)	57 (2018)
		3.1.3. Route	68	78 (2015)	60 (2016)
		3.1.4. Monitoring during anesthesia	30	48 (2016)	21 (2018)
		3.1.5. Analgesia drugs	41	56 (2014)	28 (2016)
		3.1.6. Dose of analgesia	32	39 (2015)	21 (2018)
		3.1.7. Route	24	33 (2015)	7 (2018)
		3.1.8. Surgical procedure	100	100 (All)	0 (None)
		3.1.9. Method of euthanasia	32	43 (2018)	22 (2013)
	3.2. When	3.2.1. Time of the day	0	0 (None)	0 (All)
	3.3. Where	3.3.1. Home cage	13	22 (2015)	6 (2014, 2017)
	3.4. Why	3.4.1. Choice of a specific anesthetic, route and dose	1	6 (2014)	0 (All except 2014)
4. Experimental animals	4.1. Details of animals	4.1.1. Breed	69	83 (2015)	59 (2017)
		4.1.2. Sex	58	88 (2014)	40 (2016)
		4.1.3. Age/Weight	86	100 (2014)	78 (2013)
	4.2. Further information	4.2. Source of animals	48	64 (2018)	22 (2013)
5. Housing and Husbandry	5.1. Housing	5.1.1. Type of facility	6	14 (2018)	0 (2015, 2016)
		5.1.2. Type of cage	19	33 (2015)	6 (2017)
		5.1.3. Bedding material	3	11 (2015)	0 (2013, 2014, 2016, 2017)
		5.1.4. Number of cage companions	13	31 (2014)	0 (2018)
	5.2. Husbandry conditions	5.2.1. Light/Dark cycle	9	19 (2014)	4 (2016)
		5.2.2. Temperature	10	19 (2014)	4 (2016)
		5.2.3. Humidity	6	13 (2014)	0 (2017)
		5.2.4. Type of food	36	63 (2014)	22 (2013)
		5.2.5. Access to water or food	31	56 (2014)	22 (2013)
		5.2.6. Environmental enrichment	3	11 (2015)	0 (2013, 2017, 2018)
		5.2.7. Adaptation	12	18 (2017)	4 (2016)
	5.3. Welfare related assessment	5.3.1. Welfare intervention	24	44 (2017)	12 (2015)
6. Sample size	6.1. Total number of animals used		94	100 (all except 2017)	76 (2017)
	6.2. Explanation how the number of animals was arrived at		3	6 (2015, 2016)	0 (2013, 2014, 2018)
	6.3. Indicate the number of independent replications of each experiment		11	18 (2016)	0 (2013, 2014)
7. Allocating animals to experimental groups	7.1. Details of how animals where allocated		24	38 (2014)	6 (2013)
	7.2. Order of experimental treatment		48	68 (2016)	18 (2017)
8. Experimental outcomes	8.1. Primary and secondary experimental outcomes assessed		8	13 (2014)	0 (2018)
9. Statistical methods	9.1. Details of statistical methods		76	88 (2014)	65 (2017)
	9.2. Unit of analysis for each dataset		59	72 (2015)	41 (2016)
	9.3. Methods used to assess whether the data met assumptions of the statistical approach		49	56 (2015)	41 (2016)

The data were expressed as a percentage (total mean of reports described in the totality of the articles, and averages of the maximum and the minimum of reports corresponding to the year of publication).

**Table 3:** Surgeries reported in the reviewed articles.

Type of Surgery	n of Journal	% of Journals
Craniotomies	3	2.8%
Dental surgeries	15	13.9%
Laparoscopies	28	25.9%
Laparoscopy + Thoracotomy	1	0.9%
Laparotomies	12	11.1%
Orthopedic surgeries	22	20.4%
Skin incisions	11	10.2%
Thoracotomies	16	14.8%

n: number of journals.

## Supplementary Materials

The following are available online at https://www.mdpi.com/2076-2615/9/11/947/s1, Supplementary Information S1: Studies included in the review.
