# Supplementary material for: Quality of Adherence to the ARRIVE Guidelines in the Material and Methods Section in Studies Where Swine Were Used as Surgical Biomodels: A Systematic Review (2013–2018)
Source: Animals (Basel). 2019 Nov 11;9(11):947. doi: 10.3390/ani9110947 (PMC6912630; doi:10.3390/ani9110947)
Supplement: Supplementary file 1 [file animals-09-00947-s001.pdf]

Supplementary information 1: Studies included in the review

1. Ai, L.; Liang, X.; Wang, Z.; Shen, J.; Yu, F.; Xie, L.; Pan, Y.; Lin, H. A Comparison between splenic fossa and subhepatic fossa auxiliary partial heterotopic liver transplantation in a porcine model. *Liver Transplant.* **2016**, *22*, 812–821.

2. Allegri, M.; Bugada, D.; De Gregori, M.; Avanzini, M.A.; De Silvestri, A.; Petroni, A.; Sala, A.; Filisetti, C.; Icaro Cornaglia, A.; Cobianchi, L. Continuous wound infusion with chloroprocaine in a pig model of surgical lesion: drug absorption and effects on inflammatory response. *J. Pain Res.* **2017**, *10*, 2515–2524.

3. Al-Rakan, M.; Shores, J.T.; Bonawitz, S.; Santiago, G.; Christensen, J.M.; Grant, G.; Murphy, R.J.; Basafa, E.; Armand, M.; Otovic, P.; et al. Ancillary Procedures Necessary for Translational Research in Experimental Craniomaxillofacial Surgery. *J. Craniofac. Surg.* **2014**, *25*, 2043–2050.

4. Anninga, B.; Ahmed, M.; Van Hemelrijck, M.; Pouw, J.; Westbroek, D.; Pinder, S.; ten Haken, B.; Pankhurst, Q.; Douek, M. Magnetic sentinel lymph node biopsy and localization properties of a magnetic tracer in an in vivo porcine model. *Breast Cancer Res. Treat.* **2013**, *141*, 33–42.

5. Baek, K.; Deibel, W.; Marinov, D.; Griessen, M.; Bruno, A.; Zeilhofer, H. Clinical applicability of robot-guided contact-free laser osteotomy in cranio-maxillo-facial surgery : in-vitro simulation and in-vivo surgery in minipig mandibles. *Br. J. Oral Maxillofac. Surg.* **2015**, *53*, 976–981.

6. Behrends, D.; Khendek, L.; Gao, C.; Zayed, N.; Henderson, J.; Martineau, P. Characterization of a Pre-Clinical Mini-Pig Model of Scaphoid Non-Union. *J. Funct. Biomater.* **2015**, *6*, 407–421.

7. Birck, M.; Vegge, A.; Moesgaard, S.; Eriksen, T. Single port laparoscopic long-term tube gastrostomy in Göttingen minipigs. *Lab. Anim.* **2015**, *49*, 220–227.

8. Bissinger, O.; Götz, C.; Jeschke, A.; Haller, B. Comparison of contact radiographed and stained histological sections for osseointegration analysis of dental implants : an in vivo study. *Oral Surgery, Oral Med. Oral Pathol. Oral Radiol.* **2018**, *125*, 20–26.

9. Blatnik, J.A.; Thatiparti, T.R.; Krpata, D.M.; Zuckerman, S.T.; Rosen, M.J.; von Recum, H.A. Infection prevention using affinity polymer-coated, synthetic meshes in a pig hernia model. *J. Surg. Res.* **2017**, *219*, 5–10.

10. Botzenhart, U.; Kunert-Keil, C.; Heinemann, F.; Gredes, T.; Seiler, J.; Berniczei-Roykó, Á.; Gedrange, T. Osseointegration of short titan implants: A pilot study in pigs. *Ann. Anat. - Anat. Anzeiger* **2015**, *199*, 16–22.

11. Bova, J.F.; da Cunha, A.F.; Stout, R.W.; Bhumiratana, S.; Alfi, D.M.; Eisig, S.B.; Vunjak-Novakovic, G.; Lopez, M.J. Bupivacaine Mandibular Nerve Block Affects Intraoperative Blood Pressure and Heart Rate in a Yucatan Miniature Swine Mandibular Condylectomy Model: A Pilot Study. *J. Investig. Surg.* **2015**, *28*, 32–39.

12. Caballero, M.; Morse, J.C.; Halevi, A.E.; Emodi, O.; Pharaon, M.R.; Wood, J.S.; van Aalst, J.A. Juvenile Swine Surgical Alveolar Cleft Model to Test Novel Autologous Stem Cell Therapies. *Tissue Eng. Part C Methods* **2015**, *21*, 898–908.

13. Cavallo, J.A.; Greco, S.C.; Liu, J.; Frisella, M.M.; Deeken, C.R.; Matthews, B.D.

Remodeling characteristics and biomechanical properties of a crosslinked versus a non-crosslinked porcine dermis scaffolds in a porcine model of ventral hernia repair. *Hernia* **2015**, *19*, 207–218.

14. Cervellione, R.M.; Hajnal, D.; Varga, G.; Rakoczy, G.; Kaszaki, J.; Keene, D.; Goyal, A.; Dickson, A.; Cserni, T. Mucosectomy impairs ileal microcirculation and results in flap contraction after experimental ileocystoplasty. *J. Pediatr. Urol.* **2017**, *13*, 81.e1-81.e5.

15. Chan, M.M.; Rabkin, D.G.; Washington, I.M. Clean Technique for Prolonged Nonsurvival Cardiothoracic Surgery in Swine (Sus scrofa). *J. Am. Assoc. Lab. Anim. Sci.* **2013**, *52*, 63–69.

16. Chappuis, V.; Maestre, L.; Bürki, A.; Barré, S.; Buser, D.; Zysset, P.; Bosshardt, D. Osseointegration of ultrafine-grained titanium with a hydrophilic nano-patterned surface: an in vivo examination in miniature pigs. *Biomater. Sci.* **2018**, *6*, 2448–2459.

17. Christensen, B.B.; Foldager, C.B.; Olesen, M.L.; Hede, K.C.; Lind, M. Implantation of Autologous Cartilage Chips Improves Cartilage Repair Tissue Quality in Osteochondral Defects. *Am. J. Sports Med.* **2016**, *44*, 1597–1604.

18. Coelho, P.G.; Pippenger, B.; Tovar, N.; Koopmans, S.; Plana, N.M.; Graves, D.T.; Engebretson, S.; Beusekom, H.M.M. Van; Oliveira, P.G.F.P.; Dard, M. Effect of Obesity or Metabolic Syndrome and Diabetes on Osseointegration of Dental Implants in a Miniature Swine Model : A Pilot Study. *J. Oral Maxillofac. Surg.* **2018**, *76*, 1677–1687.

19. Cserni, T.; Cervellione, R.M.; Hajnal, D.; Varga, G. Alternative ileal flap for bladder augmentation if mesentery is short. *J. Pediatr. Urol.* **2015**, *11*, 64.e1-64.e6.

20. Cui, Y.; Lu, C.; Chen, B.; Han, J.; Zhao, Y.; Xiao, Z.; Han, S.; Pan, J.; Dai, J. Restoration of mandibular bone defects with demineralized bone matrix combined with three-dimensional cultured bone marrow- derived mesenchymal stem cells in minipig models. *J. Mater. Sci. Mater. Med.* **2018**, *29*, 147.

21. Cui, Y.; Lu, C.; Meng, D.; Xiao, Z.; Hou, X.; Ding, W.; Kou, D.; Yao, Y.; Chen, B.; Zhang, Z.; et al. Collagen scaffolds modified with CNTF and bFGF promote facial nerve regeneration in minipigs. *Biomaterials* **2014**, *35*, 7819–7827.

22. Demertzis, S.; Beslac, O.; Mettler, D.; Zalokar, D.; Spangler, T.; Hausen, B.; Swanstrom, L. Beyond the "B": a new concept of the surgical staple enabling miniature staplers. *Surg. Endosc.* **2015**, *29*, 3674–3684.

23. Dolezel, R.; Ryska, O.; Kollar, M.; Juhasova, J.; Kalvach, J.; Ryska, M.; Martinek, J. A comparison of two endoscopic closures: over-the-scope clip (OTSC) versus KING closure (endoloop + clips) in a randomized long-term experimental study. *Surg. Endosc.* **2016**, *30*, 4910–4916.

24. Dubrovsky, G.; Huynh, N.; Thomas, A.; Shekherdimian, S.; Dunn, J.C.Y. Double plication for spring-mediated intestinal lengthening of a defunctionalized Roux limb ☆. *J. Pediatr. Surg.* **2018**, *53*, 1806–1810.

25. Dziewiecki, D.; van de Loo, S.; Gremse, F.; Kloss-Brandstätter, A.; Kloss, F.; Offermanns, V.; Yamauchi, K.; Kessler, P.; Lethaus, B. Osteoneogenesis due to periosteal elevation with degradable and nondegradable devices in Göttingen Minipigs. *J. Cranio-Maxillofacial Surg.* **2016**, *44*, 318–324.

26. East, B.; Kralovic, M.; Vocetkova, K.; Tonar, Z. A polypropylene mesh modified with poly- ε -caprolactone nanofibers in hernia repair : large animal experiment. *Int. J. Nanomedicine* **2018**, *13*, 3129–3143.

27. Erdogan, Ö.; Üstün, Y.; Tatli, U.; Damlar, I.; Daglıoglu, K. A Pig Model for the Histomorphometric Evaluation of Hard Tissue Around Dental Implants. *J. Oral Implantol.* **2013**, *39*, 551–557.

28. Fernandes, T.L.; Shimomura, K.; Asperti, A.; Cristina, C.; Pinheiro, G.; Vasconcellos, H.; Caetano, A.; Oliveira, C.R.G.C.M.; Nakamura, N.; Hernandez, A.J.; et al. Development of a Novel Large Animal Model to Evaluate Human Dental Pulp Stem Cells for Articular Cartilage Treatment. *Stem Cell Rev. Reports* **2018**, *14*, 734–743.

29. Fisher, M.B.; Belkin, N.S.; Milby, A.H.; Henning, E.A.; Bostrom, M.; Kim, M.; Pfeifer, C.; Meloni, G.; Dodge, G.R.; Burdick, J.A.; et al. Cartilage Repair and Subchondral Bone Remodeling in Response to Focal Lesions in a Mini-Pig Model: Implications for Tissue Engineering. *Tissue Eng. Part A* **2015**, *21*, 850–860.

30. Foerster, G.; Arnold, D.; Bischoff, S.; Boltze, K.; Harald, H.S.; Andreas, S. Pre-clinical evaluation of a minimally invasive laryngeal pacemaker system in mini-pig. *Eur. Arch. Oto-Rhino-Laryngology* **2016**, *273*, 151–158.

31. Foletti, J.; Bruneau, S.; Meningaud, J.; Berdah, S. V; Guyot, L. Endoscopic treatment of mandibular condylar fractures in live minipigs : benefits of the operative learning curve. *Br. J. Oral Maxillofac. Surg.* **2013**, *51*, 630–633.

32. Förster, G.; Arnold, D.; Bischoff, S.J.; Schubert, H.; Scholle, H.-C.; Müller, A.H. Laryngeal pacing in minipigs: in vivo test of a new minimal invasive transcricoidal electrode insertion method for functional electrical stimulation of the PCA. *Eur. Arch. Oto-Rhino-Laryngology* **2013**, *270*, 225–231.

33. Friedmann, A.; Friedmann, A.; Grize, L.; Obrecht, M.; Dard, M. Convergent methods assessing bone growth in an experimental model at dental implants in the minipig. *Ann. Anat.* **2014**, *196*, 100–107.

34. Ge, Y.; Zhang, Q.; Jiao, Z.; Li, H.; Bai, G.; Wang, H. Adipose-derived stem cells reduce liver oxidative stress and autophagy induced by ischemia-reperfusion and hepatectomy injury in swine. *Life Sci.* **2018**, *214*, 62–69.

35. Goetz, J.E.; Fredericks, D.; Petersen, E.; Rudert, M.J.; Baer, T.; Swanson, E.; Roberts, N.; Martin, J.; Tochigi, Y. A clinically realistic large animal model of intra-articular fracture that progresses to post-traumatic osteoarthritis. *Osteoarthr. Cartil.* **2015**, *23*, 1797–1805.

36. Ortega, A.; Roca, A.; Micó, J.A. Modelos animales de dolor. Una visión crítica. *Rev. la Soc. Esp. del Dolor* **2002**, *9*, 447–453.

37. Guo, J.; Sun, B.; Sun, S.; Liu, X.; Wang, S.; Ge, N.; Wang, G.; Liu, W. Endoscopic puncture-suture device to close gastric wall defects after full-thickness resection: a porcine study. *Gastrointest. Endosc.* **2017**, *85*, 447–450.

38. Heinke, S.; Ludwig, B.; Schubert, U.; Schmid, J.; Kiss, T.; Steffen, A.; Bornstein, S.; Ludwig, S. Diabetes induction by total pancreatectomy in minipigs with simultaneous splenectomy: a feasible approach for advanced diabetes research. *Xenotransplantation* **2016**, *23*, 405–413.

39. Henlin, T.; Michalek, P.; Tyll, T.; Ryska, O. A Randomized Comparison of Bougie-Assisted and TracheoQuick Plus Cricothyrotomies on a Live Porcine Model. *Biomed Res. Int.* **2017**, *2017*, 1–6.

40. Hernández Hurtado, L.; Sánchez-Margallo, F.M.; De la Cruz Vigo, J.L.; Maestre Antequera, J.; Matos Azevedo, A.M.; Casado, J.G.; Díaz-Güemes Martín-Portugués, I. Changes on Adipose Tissue Distribution After Laparoscopic Roux-en-Y Gastric Bypass in Obese Göttingen Minipig. Effects on Glucose Metabolism. *Obes. Surg.* **2016**, *26*, 3001–3006.

41. Hsu, H.C.; Enosawa, S.; Yamazaki, T.; Tohyama, S.; Fujita, J.; Fukuda, K.; Kobayashi, E. Enhancing Survival of Human Hepatocytes by Neonatal Thymectomy and Partial Hepatectomy in Micro-miniature Pigs. *Transplant. Proc.* **2017**, *49*, 153–158.

42. Huang, M.; Chen, L.; Ou, K.-L.; Cheng, H.; Wang, C. Rapid Osseointegration of Titanium Implant With Innovative Nanoporous Surface Modification: Animal Model and Clinical Trial. *Implant Dent.* **2015**, *0*, 1–7.

43. Iguchi, K.; Hatano, E.; Yamanaka, K.; Sato, M.; Yamamoto, G.; Kasai, Y.; Okamoto, T.; Okuno, M.; Taura, K.; Fukumoto, K.; et al. Hepatoprotective effect by pretreatment with olprinone in a swine partial hepatectomy model. *Liver Transplant.* **2014**, *20*, 838–849.

44. Ioannou, C. V; Stergiopulos, N.; Georgakarakos, E.; Chatzimichali, E.; Katsamouris, A.N.; Morel, D.R. Effects of Isoflurane Anesthesia on Aortic Compliance and Systemic Hemodynamics in Compliant and Noncompliant Aortas. *J. Cardiothorac. Vasc. Anesth.* **2013**, *27*, 1282–1288.

45. Irvine, K.; Bishop, R.K.; Won, S.J.; Xu, J.; Hamel, K.A.; Coppes, V.; Singh, P.; Sondag, A.; Rome, E.; Basu, J.; et al. Effects of Veliparib on Microglial Activation and Functional Outcomes after Traumatic Brain Injury in the Rat and Pig. *J. Neurotrauma* **2017**.

46. Ishikawa, O.; Tanaka, M.; Konno, K.; Hasebe, T. Swine model of in-stent stenosis in the iliac artery evaluating the serial time course. *Exp. Anim.* **2018**, *67*, 501–508.

47. Itoda, Y.; Panthee, N.; Tanaka, T. Novel Anastomotic Device for Distal Coronary Anastomosis : Preclinical Results From Swine Off-Pump Coronary Artery Bypass Model. *Ann. Thorac. Surg.* **2016**, *101*, 736–741.

48. Jagodzinski, M.; Liu, C.; Guenther, D.; Burssens, A.; Petri, M.; Abedian, R.; Willbold, E.; Krettek, C.; Haasper, C.; Witte, F. Bone Marrow-Derived Cell Concentrates Have Limited Effects on Osteochondral Reconstructions in the Mini Pig. *Tissue Eng. Part C Methods* **2014**, *20*, 215–226.

49. Jensen, H.; Jensen, M.O.; Waziri, F.; Honge, J.L.; Sloth, E.; Fenger-Gron, M.; Nielsen, S.L. Transapical neochord implantation: Is tension of artificial chordae tendineae dependent on the insertion site? *J. Thorac. Cardiovasc. Surg.* **2014**, *148*, 138–143.

50. Kiapour, A.M.; Fleming, B.C.; Proffen, B.L.; Murray, M.M. Sex Influences the Biomechanical Outcomes of Anterior Cruciate Ligament Reconstruction in a Preclinical Large Animal Model. *Am. J. Sports Med.* **2015**, *43*, 1623–1631.

51. Kim, D.-Y.; Kim, J.-R.; Jang, K.Y.; Kim, M.G.; Lee, K. Evaluation of Titanium-Coated Pedicle Screws: In Vivo Porcine Lumbar Spine Model. *World Neurosurg.* **2016**, *91*, 163–171.

52. Kim, I.L.; Pfeifer, C.G.; Fisher, M.B.; Saxena, V.; Meloni, G.R.; Kwon, M.Y.; Kim, M.; Steinberg, D.R.; Mauck, R.L.; Burdick, J.A. Fibrous Scaffolds with Varied Fiber Chemistry and Growth Factor Delivery Promote Repair in a Porcine Cartilage Defect Model. *Tissue Eng. Part A* **2015**, *21*, 2680–2690.

53. Kotsougiani, D.; Hundepool, C.A.; Bulstra, L.F.; Friedrich, P.F.; Shin, A.Y.; Bishop, A.T. Recipient-derived angiogenesis with short term immunosuppression increases bone remodeling in bone vascularized composite allotransplantation: A pilot study in a swine tibial defect model. *J. Orthop. Res.* **2016**, *35*, 1242–1249.

54. Kotsougiani, D.; Willems, J.I.; Shin, A.Y.; Friedrich, P.F.; Hundepool, C.A.; Bishop, A.T. A new porcine vascularized tibial bone allotransplantation model. Anatomy and surgical technique. *Microsurgery* **2017**, *38*, 195–202.

55. Krüger, M.; Zinne, N.; Biancosino, C.; Höffler, K.; Rajab, T.K.; Waldmann, K.-H.; Jonigk, D.; Avsar, M.; Haverich, A.; Hoeltig, D. Porcine pulmonary auto-transplantation for ex vivo therapy as a model for new treatment strategies. *Interact. Cardiovasc. Thorac. Surg.* **2016**, *23*, 358–366.

56. Leng, J.; Xing, H.; Tan, J.; Chen, K.; Dong, J. The Safe Minimally Ischemic Liver Remnant for Small-for-Size Syndrome in Porcine Hepatectomy. *Transplant. Proc.* **2013**, *45*, 2419–2424.

57. Leto Barone, A.A.; Leonard, D.A.; Torabi, R.; Mallard, C.; Glor, T.; Scalea, J.R.; Randolph, M.A.; Sachs, D.H.; Cetrulo, C.L. The gracilis myocutaneous free flap in swine: An advantageous preclinical model for vascularized composite allograft transplantation research. *Microsurgery* **2013**, *33*, 51–55.

58. Lin, T.; Hu, H.; Wang, H.; Wu, M.-C.; Wu, S.; Yeh, M.-L. Evaluation of osseous integration of titanium orthopedic screws with novel SLA treatment in porcine model. *PLoS One* **2017**, *12*, e0188364.

59. Liñares, A.; Domken, O.; Dard, M.; Blanco, J. Peri-implant soft tissues around implants with a modified neck surface. Part 1. Clinical and histometric outcomes: a pilot study in minipigs. *J. Clin. Periodontol.* **2013**, *40*, 412–420.

60. Liu, S.; Liu, Z.; Li, L.; Liu, P.; Liu, H. Keeping the heart empty and beating: an alternative technique to preserve hypertrophied hearts during valvular surgery. *J. Cardiothorac. Surg.* **2015**, *10*, 71.

61. Liu, W.; Tang, X.; Zhang, Z.; Yin, L.; Gui, L. 3D-CT evaluation of mandibular morphology after mandibular outer cortex osteotomy in young miniature pigs: The role of the periosteum. *J. Cranio-Maxillofacial Surg.* **2013**, *42*, 1–9.

62. Liu, X.; Yang, Y.; Meng, Q.; Sun, J.; Luo, F.; Cui, Y.; Zhang, H.; Zhang, D.; Tang, Y. A Secure and High-Fidelity Live Animal Model for Off-Pump Coronary Bypass Surgery Training. *J. Surg. Educ.* **2016**, *73*, 583–588.

63. Liu, Y.; Wang, J.; Yang, P.; Lu, H.; Lu, L.; Wang, J.; Li, H.; Duan, Y.; Wang, J.; Li, Y. Delayed rearterialization unlikely leads to nonanastomotic stricture but causes temporary injury on bile duct after liver transplantation. *Transpl. Int.* **2015**, *28*, 341–351.

64. Lohan, A.; Marzahn, U.; El Sayed, K.; Bock, C.; Haisch, A.; Kohl, B.; Stoelzel, K.; John, T.; Ertel, W.; Schulze-Tanzil, G. Heterotopic and orthotopic autologous chondrocyte implantation using a minipig chondral defect model. *Ann. Anat. - Anat. Anzeiger* **2013**,

195, 488–497.

65. Ma, L.; Cai, X.; Wang, H.; Yu, Y.-L.; Huang, D.; Ge, G.; Hu, H.; Yu, S. Laparoscopic colonic anastomosis using a degradable stent in a porcine model. *World J. Gastroenterol.* **2016**, *22*, 4707–4715.

66. Madariaga, M.L.L.; Spencer, P.J.; Michel, S.G.; La Muraglia, G.M.; O'Neil, M.J.; Mannon, E.C.; Leblang, C.; Rosales, I.A.; Colvin, R.B.; Sachs, D.H.; et al. Effects of Lung Cotransplantation on Cardiac Allograft Tolerance Across a Full Major Histocompatibility Complex Barrier in Miniature Swine. *Am. J. Transplant.* **2016**, *16*, 979–986.

67. Marchini, G.S.; Júniori, I.D.F.; Horta, L. V; Torricelli, F.C.M.; Mitre, A.I.; Arap, M.A. Specific training for LESS surgery results from a prospective study in the animal model. *Int. braz J Urol* **2016**, *42*, 90–95.

68. McKenney, M.L.; Schultz, K.A.; Boyd, J.H.; Byrd, J.P.; Alloosh, M.; Teague, S.D.; Arce-Esquivel, A.A.; Fain, J.N.; Laughlin, M.H.; Sacks, H.S.; et al. Epicardial adipose excision slows the progression of porcine coronary atherosclerosis. *J. Cardiothorac. Surg.* **2014**, *9*, 2.

69. Mehl, C.; Kern, M.; Neumann, F.; Bähr, T.; Wiltfang, J.; Gassling, V. Effect of ultraviolet photofunctionalization of dental titanium implants on osseointegration *. *J. Zhejiang Univ. B (Biomedicine Biotechnol.* **2018**, *19*, 525–534.

70. Miura, K.; Sahara, H.; Waki, S.; Kawai, A.; Sekijima, M.; Kobayashi, T.; Zhang, Z.; Wakai, T.; Shimizu, A.; Yamada, K. Development of the Intestinal Transplantation Model With Major Histocompatibility Complex Inbred CLAWN Miniature Swine. *Transplant. Proc.* **2016**, *48*, 1315–1319.

71. Morillas-Sendín, P.; Delgado-Baeza, E.; Delgado-Martos, M.J.; Barranco, M.; del Cañizo, J.F.; Ruíz, M.; Quintana-Villamandos, B. Effects of Sevoflurane and Propofol on Organ Blood Flow in Left Ventricular Assist Devices in Pigs. *Biomed Res. Int.* **2015**, 1–9.

72. Olver, T.D.; Hiemstra, J.A.; Edwards, J.C.; Ferguson, B.S.; Laughlin, M.H.; Emter, C.A. The protective role of sex hormones in females and exercise prehabilitation in males on sternotomy-induced cranial hypoperfusion in aortic banded mini-swine. *J. Appl. Physiol.* **2017**, *122*, 423–429.

73. Pepper, A.R.; Welch, I.; Bruni, A.; MacGillivary, A.; Mazzuca, D.M.; White, D.J.G.; Wall, W. Establishment of a Stringent Large Animal Model of Insulin-Dependent Diabetes for Islet Autotransplantation. *Pancreas* **2013**, *42*, 329–338.

74. Popa, F.; Georgescu, A. V Abdominal Wall Reconstruction after Flap Surgery and the Effect on the Immune System. *Biomed Res. Int.* **2017**, 1–10.

75. Ramot, Y.; Rousselle, S.D.; Yellin, N.; Willenz, U.; Sabag, I.; Avner, A.; Nyska, A. Biocompatibility and Systemic Safety of a Novel Implantable Annuloplasty Ring for the Treatment of Mitral Regurgitation in a Minipig Model. *Toxicol. Pathol.* **2016**, *44*, 655–662.

76. Ríos-Santos, J. V., A.M.M.-G.M.H.-C.B.R.-C.A.F.-P.; Perez, R.A.; Gil, F.J. Unravelling the effect of macro and microscopic design of dental implants on osseointegration : a randomised clinical study in minipigs. *J. Mater. Sci. Mater. Med.* **2018**, *29*, 99.

77. Ryou, M.; Aihara, H.; Thompson, C.C. Minimally invasive entero-enteral dual-path bypass using self-assembling magnets. *Surg. Endosc.* **2016**, *30*, 4533–4538.

78. Sang, J.; Shi, X.; Han, B.; Huang, X.; Huang, T.; Ren, H.; Ding, Y. Combined mesenchymal stem cell transplantation and interleukin-1 receptor antagonism after partial hepatectomy. *World J. Gastroenterol.* **2016**, *22*, 4120–4135.

79. Sasaki, R.; Matsumine, H.; Watanabe, Y.; Yamato, M.; Ando, T. Surgical procedure of extracting teeth for obtaining dental pulp for regenerative medicine in swine. *Lab. Anim.* **2015**, *49*, 172–176.

80. Schaller, B.; Saulacic, N.; Imwinkelried, T.; Beck, S.; Wei, E.; Liu, Y.; Gralla, J.; Nakahara, K.; Hofstetter, W.; Iizuka, T. In vivo degradation of magnesium plate / screw osteosynthesis implant systems : Soft and hard tissue response in a calvarial model in miniature pigs. *J. Cranio-Maxillofacial Surg.* **2016**, *44*, 309–317.

81. Schilling, T.; Brandes, G.; Tudorache, I.; Cebotari, S.; Hilfiker, A.; Meyer, T.; Biskup, C.; Bauer, M.; Waldmann, K.; Bach, F.; et al. In vivo degradation of magnesium alloy LA63 scaffolds for temporary stabilization of biological myocardial grafts in a swine model. *Biomed. Tech. Eng.* **2013**, *58*, 407–416.

82. Sham, J.G.; Simianu, V. V; Wright, A.S.; Stewart, S.D.; Alloosh, M.; Sturek, M.; Cummings, D.E.; Flum, D.R. Evaluating the Mechanisms of Improved Glucose Homeostasis after Bariatric Surgery in Ossabaw Miniature Swine. *J. Diabetes Res.* **2014**, *2014*, 1–7.

83. Sheu, S.Y.; Wang, C.H.; Pao, Y.H.; Fu, Y.T.; Liu, C.H.; Yao, C.H.; Kuo, T.F. The effect of platelet-rich fibrin on autologous osteochondral transplantation: An in vivo porcine model. *Knee* **2017**, *24*, 1392–1401.

84. Sivan-Gildor, A.; Machtei, E.E.; Gabay, E.; Frankenthal, S.; Levin, L.; Suzuki, M.; Coelho, P.G.; Zigdon-Giladi, H. Novel Implant Design Improves Implant Survival in Multirooted Extraction Sites: A Preclinical Pilot Study. *J. Periodontol.* **2014**, *85*, 1458–1463.

85. Smith, S.; McCully, B.; Bommiasamy, A.; Murphy, J.; Behrens, B.; Pati, S.; Goodman, A.; Schreiber, M. A Combat Relevant Model for the Creation of Acute Lung Injury in Swine. *J. Trauma Acute Care Surg.* **2017**, 1–21.

86. Song, T.J.; Seo, D.W.; Kim, S.H.; Park, D.H.; Lee, S.S.; Lee, S.K.; Kim, M.H. Endoscopic gastrojejunostomy with a natural orifice transluminal endoscopic surgery technique. *World J. Gastroenterol.* **2013**, *19*, 3447.

87. Sterkers, A.; Hubert, T.; Gmyr, V.; Torres, F.; Baud, G.; Delalleau, N.; Vantyghem, M.C.; Kerr-Conte, J.; Caiazzo, R.; Pattou, F. Islet Survival and Function Following Intramuscular Autotransplantation in the Minipig. *Am. J. Transplant.* **2013**, *13*, 891–898.

88. Stricker, A.; Fleiner, J.; Dard, M.; Voss, P.; Sauerbier, S.; Bosshardt, D.D. Evaluation of a new experimental model to study bone healing after ridge expansion with simultaneous implant placement - a pilot study in minipigs. *Clin. Oral Implants Res.* **2014**, *25*, 1265–1272.

89. Stricker, A.; Fleiner, J.; Stübinger, S.; Fleiner, H.; Buser, D.; Bosshardt, D.D. Ridge preservation after ridge expansion with simultaneous guided bone regeneration: a preclinical study. *Clin. Oral Implants Res.* **2016**, *27*, e116–e124.

90. Sullins, V.F.; Traum, P.K.; French, S.W.; Wu, B.M.; Dunn, J.C.Y.; Lee, S.L. A novel method of esophageal lengthening in a large animal model of long gap esophageal atresia. *J. Pediatr. Surg.* **2015**, *50*, 928–932.

91. Suzuki, T.; Kawamoto, S.; Nakagawa, A.; Endo, T.; Tominaga, T.; Akiyama, M.; Adachi, O.; Kumagai, K.; Saiki, Y. Application of actuator-driven pulsed water jet for coronary artery bypass grafting: assessment in a swine model. *J. Artif. Organs* **2018**, *21*, 247–253.

92. Vallabhajosyula, P.; Hirakata, A.; Weiss, M.; Griesemer, A.; Shimizu, A.; Hong, H.; Habertheuer, A.; Tchipashvili, V.; Yamada, K.; Sachs, D.H. Effect of the Diabetic State on Islet Engraftment and Function in a Large Animal Model of Islet–Kidney Transplantation. *Cell Transplant.* **2017**, *26*, 1755–1762.

93. Verhaeghe, R.; Zerrweck, C.; Hubert, T.; Tréchot, B.; Gmyr, V.; D'Herbomez, M.; Pigny, P.; Pattou, F.; Caiazzo, R. Gastric Bypass Increases Postprandial Insulin and GLP-1 in Nonobese Minipigs. *Eur. Surg. Res.* **2014**, *52*, 41–49.

94. Verket, A.; Lyngstadaas, S.P.; Tiainen, H.; Rønold, H.J.; Impact, J.C.W. Impact of particulate deproteinized bovine bone mineral and porous titanium granules on early stability and osseointegration of dental implants in narrow marginal circumferential bone defects. *Int. J. Oral Maxillofac. Surg.* **2018**, *47*, 1086–1094.

95. Wagner, R.; Piler, P.; Uchytil, B.; Halouzka, R.; Kovaru, H.; Bobkova, M.; Nemec, P. Systemic inflammatory response syndrome is reduced by preoperative plasma-thrombo-leukocyte aphaeresis in a pig model of cardiopulmonary bypass. *Biomed. Pap.* **2016**, *160*, 399–406.

96. Wang, D.; Xu, Y.; Zhu, Z.; Tan, X.; Tu, Y.; Han, M.; Tan, J.-W. Should temporary extracorporeal continuous portal diversion replace meso/porta-caval shunts in "small-for-size" syndrome in porcine hepatectomy? *World J. Gastroenterol.* **2015**, *21*, 888–896.

97. Willens, S.; Cox, D.M.; Braue, E.H.; Myers, T.M.; Wegner, M.D. Novel Technique for Retroperitoneal Implantation of Telemetry Transmitters for Physiologic Monitoring in Göttingen Minipigs (Sus scrofa domesticus). *Comp. Med.* **2014**, *64*, 464–470.

98. Wu, D.-B.; Yang, S.-F.; Geng, K.-H.; Qin, S.-J.; Bao, Y.-L.; Chen, X.; Zheng, G.-P. Preliminary Study on the Application of an Umbrella-Like Abdominal Wall-Lifting Device in Gasless Laparoscopic Surgery. *J. Laparoendosc. Adv. Surg. Tech.* **2013**, *23*, 246–249.

99. Xue, J.; He, A.; Zhu, Y.; Liu, Y.; Li, D.; Yin, Z.; Zhang, W.; Liu, W.; Cao, Y.; Zhou, G. Repair of articular cartilage defects with acellular cartilage sheets in a swine model. *Biomed. Mater.* **2017**, *13*, 025016.

100. Yao, C; Hedrick, M; Pareek, G; Renzulli, J; Haleblian, G; Webster, T. Nanostructured polyurethane-poly-lactic- co-glycolic acid scaffolds increase bladder tissue regeneration: an in vivo study. *Int. J. Nanomedicine* **2013**, *8*, 3285–3296.

101. Ye, W.; Duan, Y.Z.; Liu, Z.J. Alteration of functional loads after tongue volume reduction. *Orthod. Craniofac. Res.* **2013**, *16*, 234–245.

102. Young, D.A.; Jackson, N.; Ronaghan, C.A.; Brathwaite, C.E.M.; Gilbert, T.W. Retrorectus repair of incisional ventral hernia with urinary bladder matrix reinforcement in a long-term porcine model. *Regen. Med.* **2018**, *13*, 395–408.

103. Yu-Liang Tu, Xuan Wang, Da-Dong Wang, Zi-Man Zhu, J.-W.T.; Yu-Liang Impact of mesocaval shunt on safe minimal liver remnant: Porcine model. *World J. Gastroenterol.* **2013**, *19*, 5076–5084.

104. Zhang, W.; Weng, G.; Li, M.; Yu, S.; Bao, J.; Cao, X.; Dou, Z.; Wang, H.; Chen, H. Original Research: Establishment of an early embolus-related cerebral injury model after cardiopulmonary bypass in miniature pigs. *Exp. Biol. Med.* **2016**, *241*, 1819–1824.

105. Zhang, X.; Liu, J.; Wu, Q.; Liu, Z.; Yan, Z. Uterus Allo-Transplantation in a Swine Model: Long-Term Graft Survival and Reproductive Function. *Med. Sci. Monit.* **2018**, *24*, 8422–8429.

106. Zhong, H.; Wang, Z.; Yang, Z.; Zhao, F.; Wang, B.; Liu, P. CO2 laser soldering for the reconstruction of dural defect in the minipig model. *Turk. Neurosurg.* **2012**, *26*, 240–245.

107. Zhou, W.; Lin, L.; Cheng, Y.; Liu, Y. Ursolic Acid Improves Liver Transplantation and Inhibits Apoptosis in Miniature Pigs Using Donation After Cardiac Death. *Cell. Physiol. Biochem.* **2017**, *43*, 331–338.

108. Zhou, W.; Wang, X.; He, Y.; Nie, Y.; Zhang, G.; Wang, C.; Wang, C.; Wang, X. N - 11 C-Methyl-Dopamine PET Imaging of Sympathetic Nerve Injury in a Swine Model of Acute Myocardial Ischemia: A Comparison with 13 N-Ammonia PET. *Biomed Res. Int.* **2016**, *2016*, 1–8.
